# Are children with clinical obesity at increased risk of inpatient hospital admissions? An analysis using linked electronic health records in the UK millennium cohort study

**DOI:** 10.1111/ijpo.12505

**Published:** 2019-01-18

**Authors:** Lucy J. Griffiths, Mario Cortina‐Borja, Amrita Bandyopadhyay, Karen Tingay, Bianca L. De Stavola, Helen Bedford, Ashley Akbari, Nicola Firman, Ronan A. Lyons, Carol Dezateux

**Affiliations:** ^1^ Health Data Research UK, Wales and Northern Ireland Swansea University Medical School Swansea UK; ^2^ Life Course Epidemiology and Biostatistics UCL Great Ormond Street Institute of Child Health London UK; ^3^ Clinical Epidemiology, Nutrition and Biostatistics UCL Great Ormond Street Institute of Child Health London UK; ^4^ National Centre for Population Health and Wellbeing Research Swansea University Medical School Swansea UK; ^5^ Administrative Data Research Centre Wales Swansea University Medical School Swansea UK; ^6^ Centre for Primary Care and Public Health, Barts and the London School of Medicine and Dentistry Queen Mary University of London London UK; ^7^ Health Data Research UK London Queen Mary University London London UK

**Keywords:** cohort study, health service utilization, obesity, record linkage

## Abstract

**Background:**

Few studies have examined health service utilization of children with overweight or obesity by using linked electronic health records (EHRs).

**Objective/Methods:**

We analysed EHRs from 3269 children (1678 boys; 51.3% [weighted]) participating in the Millennium Cohort Study, living in Wales or Scotland at age seven whose parents consented to record linkage. We used height and weight measurements at age five to categorize children as obese (>98th centile) or overweight (>91st centile) (UK1990 clinical reference standards) and linked to hospital admissions, up to age 14 years, in the Patient Episode Database for Wales and Scottish Morbidity Records. Negative binomial regression models compared rates of inpatient admissions by weight status at age five.

**Results:**

At age five, 11.5% and 6.7% of children were overweight or obese, respectively; 1221 (38%) children were subsequently admitted to hospital at least once. Admissions were not increased among children with overweight or obesity (adjusted rate ratio [RR], 95% confidence interval [CI]: 0.87, 0.68‐1.10 and 1.16, 0.87‐1.54, respectively).

**Conclusions:**

In this nationally representative cohort of children in Wales and Scotland, those with overweight or obesity at entry to primary school did not have increased rates of hospital admissions in later childhood and early adolescence.

## 1 INTRODUCTION

Obesity in children and young people (CYP) continues to pose a significant public health challenge. Analyses of data from four nationally representative UK birth cohort studies show that, compared with earlier generations, the probability of childhood overweight or obesity is two to three times greater than among those born after the 1980s.^1^ Currently, one in five children is overweight or obese during their first year of primary school in England, Scotland, and Wales.^2^ Data from the 2016/17 National Child Measurement Programme (NCMP) in England suggests that obesity has increased in this year group since 2015/16; furthermore, inequalities in obesity continue to widen between the most deprived and least deprived children.^3^

There are well‐established implications of paediatric obesity in the short term for CYP's physical health, development, and well‐being^4^ and, in the longer term, for health in adult life. These “potentially devastating” consequences,^5^ coupled with the high prevalence of this condition, pose a potentially significant burden on community, primary, and secondary health services.

While obesity in CYP is associated with increased health care costs,^6^, ^7^, ^8^, ^9^, ^10^, ^11^ the association with long‐term, or different types of, health care utilization is less clear. The majority of studies investigating this issue are from the United States (US) and may not translate to other settings; furthermore, the majority have explored accident and emergency (A&E) department attendances and largely report that children with overweight or obesity use these services more than their healthy weight peers.^12^, ^13^ Studies of primary care consultations and hospital admissions are fewer and less consistent.^8^, ^12^, ^14^ However, UK hospital discharge records suggest that hospital admissions coded for obesity, and related comorbid conditions, have increased more than fourfold over the past decade among CYP in England^15^; these have also increased in the Republic of Ireland.^16^ In a recent report, Viner and colleagues identified discordance between the population burden of obesity and available information on health service use in CYP.^17^

To date, there have been few reports of analyses that have examined weight status at the start of primary school (which is around 5 years of age for most children in the United Kingdom) in relation to subsequent health service utilization through primary school and early adolescence. This is the age when public health surveillance data on child weight status is widely collected,^3^, ^18^, ^19^ given the importance of early detection of children who may be on unhealthy weight trajectories^20^, ^21^ and in need of support to facilitate a healthier lifestyle. Hence, the aim of this study was to link data from the Millennium Cohort Study (MCS), currently the largest contemporary and UK representative child cohort study with prospective anthropometric measures, with electronic health records (EHRs) from Wales and Scotland, to examine the impact of childhood obesity and overweight on subsequent hospital admissions. We hypothesized that overweight or obesity status by age 5 years would be associated with increased risk of hospital admissions during later childhood and early adolescence.

## 2 METHODS

### 2.1 Study population

We used data from the MCS, a prospective study of children born between September 2000 and January 2002 in the United Kingdom. Infants who were alive and living in the United Kingdom at age 9 months were sampled from child benefit registers. Disproportionately stratified sampling at electoral ward level ensured adequate representation of disadvantaged and ethnic minority areas. Further information on the cohort and sampling design can be found in the MCS cohort profile.^22^

The original cohort comprised 18 819 children (2799 in Wales and 2370 in Scotland) whose carers (including, usually, the child's mother) were first interviewed at home when their child was aged 9 months (known as MCS1). Five further home interviews have been administered to date, at ages three (MCS2), five (MCS3), seven (MCS4), eleven (MCS5), and fourteen (MCS6) years. At each survey, information has been collected on demographic, social, and health factors (including anthropometric data) related to the child and their family. At MCS4, adults with parental responsibility were also asked to consent to link information collected within MCS to their child's routine health records up until their 14th birthday. Of the 13 681 singletons who participated in this survey, 1951 and 1598 were interviewed in Wales and Scotland, respectively; consent for record linkage was obtained for a total of 3304 of these children (1839 in Wales and 1465 in Scotland) (93% of those interviewed in these nations). While consent was requested for the whole MCS cohort across the United Kingdom, this study was restricted to those interviewed within these two nations because of linkage difficulties and delays in England and Northern Ireland within the period for which we had study funding.

### 2.2 Record linkage

**Figure 1:**
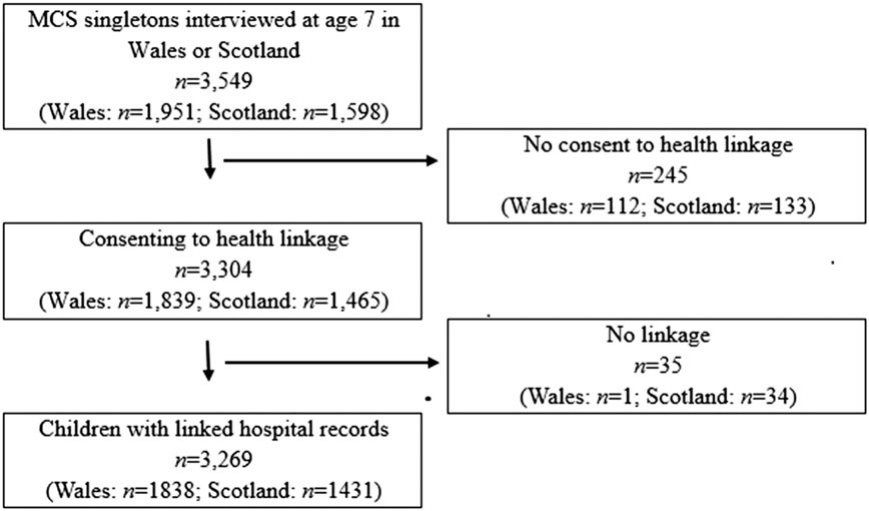
Flow diagram of participants

Linkage of EHRs to MCS data was carried out in Scotland by the National Health Service (NHS) Information Standards Division (ISD) and in Wales by assigning unique Anonymized Linkage Fields to person‐based records in the privacy protecting Secure Anonymised Information Linkage (SAIL) Databank at Swansea University.^23^, ^24^, ^25^ Figure 1 highlights the process for obtaining the study population; linkage was achieved for 98.9% of the singletons (*n* = 3269) whose parents provided consent.

### 2.3 Exposure variable

Of those who provided consent for record linkage at MCS4, we derived our weight status exposure variable from information collected at MCS3 (5 years) as follows: Trained interviewers weighed the children, without shoes or outdoor clothing, using Tanita HD‐305 scales (Tanita UK Ltd., Middlesex, United Kingdom) and weight recorded in kilograms to one decimal place. Height was also measured with the Leicester Height Measure Stadiometer (Seca Ltd., Birmingham, United Kingdom) and recorded to the nearest millimetre. Body mass index (BMI) (weight [kg]/height [m]^2^) was categorized according to the UK1990 clinical reference standard,^26^ into four mutually exclusive groups: “underweight” (BMI <second centile), “healthy weight” (≥second to <91st centile), “overweight” (≥91st to <98th centile), or “obese” (≥98th centile) based on alignment with sex‐ and age‐specific BMI centiles from the LMS growth tool Excel add‐in.^27^, ^28^ Given that only 0.5% (weighted estimate on imputed sample) were classified as underweight, this group of children was combined with the healthy weight group (whole group hereafter termed “healthy weight”).

### 2.4 Outcome variable

We identified all hospital admissions in these children between the ages of five and 13.99 years in the linked Patient Episode Database for Wales (PEDW) and the linked Scottish Morbidity Records (SMR). We defined a hospital admission as a distinct continuous inpatient spell (CIP), also known as a person super spell in SAIL; this is a definitive period of care within the NHS from admission to hospital to discharge (or death). It may contain one or more provider (organization) spells, which may, in turn, contain one or more episodes (a period of care under one consultant). Readmissions within 48 hours were included in the same CIP. For each child, we counted the total number of CIPs during the defined eight‐year follow‐up period.

### 2.5 Potential confounders

We explored a range of potential confounders that were identified in previous studies as being related to overweight and/or to hospital admissions. The following potential confounding factors were examined: child: sex (MCS1), mode of delivery (MCS1), preterm (<37 weeks) delivery (MCS1), long‐standing illness (main survey respondents report of any illness, disability or infirmity affecting the child over a period of time), average hours of screen time; maternal: weight status (BMI (kg/m2): underweight: <18.5, healthy weight: 18.5–24.9, overweight: 25‐29.9, obese: ≥30), highest academic qualification (MCS1‐3), lone parenthood; household: smoking in the same room as cohort child, number of people in the household, household income (<60% of the national median using a modified Organization for Economic Cooperation and Development [OECD] equivalized scale); area‐level: 2005 Rural/Urban Area Classification (RUAC). These were all measured at age 5 years (MCS3), except where indicated.

### 2.6 Statistical methods

As 62% of children had no admissions, and some children had a relatively large number of admissions (implying that the variance was larger than the mean), zero‐inflated negative binomial (ZINB) models were initially considered to model the latent processes generating the observed number of admissions. However, the fitted ZINB models that included multiple imputation and survey weights in the estimation procedure had numeric problems and produced unfeasible standard errors. Thus, we fitted negative binomial regression (NBR) models^29^ to explain over dispersion in the count data. These models accounted appropriately for the large proportion of zeroes. We estimated unadjusted and adjusted rate ratios (RRs) for hospital admissions from age five to 13.99 years, for overweight or obesity at age five, with healthy weight status as baseline. In all regression models, data from two children with over 40 hospital admissions were excluded from the analyses. Adjusted models included those covariates that were statistically significant (according to *F* tests for goodness of fit) in the unadjusted models. Adjusted models were also run with and without long‐standing illness. Analyses were also carried out to examine this relationship with secondary outcomes: hospital admissions from five to 9.99 years and 10‐13.99 years.

We then explored reasons for admissions based on a prespecified list of International Classification of Diseases (ICD10) diagnosis codes (Appendix 1). The most common reasons for admission (>200 children admitted) were examined in relation to weight status at age five; we derived the weighted proportion of children with these types of admissions by weight status at age five, and fitted unadjusted NBR models to obtain *F* tests of goodness of fit (adjusted models were not fitted because of small‐group sizes).

Multiple imputation was performed to estimate missing height and weight values at age five (*n* = 193) and missing data for covariates within the models. Twenty imputed data sets were built using the weighted iterative chain algorithm, including all variables involved in the analysis steps including hospital admissions under the assumption that missingness is at random (MAR).^30^ Complete case analyses were also conducted, although all results are based on the analyses with imputed data unless indicated.

Analyses were performed using STATA/SE 15.0 (Stata Corporation, Texas) using survey and non‐response weights to account for the clustered sampling, attrition between contacts, and consent to data linkage.^31^

## 3 RESULTS

Most children (97.4%) were White, 51.3% were boys, and 18.1% had a reported long‐standing illness at age five. Using UK1990 clinical cut‐offs, 11.2% of children were overweight, and 6.4% were obese at age five with 82.5% of the children defined as being of healthy weight. Following imputation, these estimates changed marginally, with 11.5%, 6.7%, and 81.7% classified as overweight, obese, and of healthy weight, respectively. Other individual and household characteristics are summarized in Table 1.

**Table 1:** Sample characteristics

	*n* (weighted %) *N* = 3269
Sex	
Male	1678 (51.3)
Female	1591 (48.7)
Child's ethnicity	
White ethnic group	3181 (97.4)
All other groups	83 (2.6)
Longstanding illness of child	
No	2521 (81.9)
Yes	588 (18.1)
Child's weight status*	
Healthy weight	2522 (82.5)
Overweight	352 (11.2)
Obese	202 (6.4)
Mother's weight status	
Underweight	102 (3.5)
Healthy weight	1377 (41.6)
Overweight	726 (21.3)
Obese	1064 (33.6)
Maternal highest academic qualification**	
Degree	651 (18.8)
HE diploma	348 (9.5)
A/AS/S levels	413 (14.3)
O level/GCSE	1328 (40.6)
Other	81 (2.4)
None	438 (14.4)
Lone parent or carer	
Non‐lone	2533 (79.6)
Lone	586 (20.4)
Smoking in the same room	
No	2587 (81.4)
Yes	521 (18.6)
Number of people in the household (incl. cohort child**)**	
2	169 (6.4)
3	564 (18.3)
4	1420 (43.9)
5 or more people	966 (31.4)
OECD 60% median poverty indicator	
Above	2213 (69.1)
Below	894 (30.9)
Living area	
Urban	2360 (73.9)
Rural	756 (26.1)

Table footnotes:

Missing data arising from item non‐response (NR) and non‐participation in MCS3 (NP): ethnicity (NR: 5); long‐standing illness of child (NR: 10; NP: 150); weight status of child (NR: 43; NP: 150); maternal highest academic qualification (NR: 10); lone parent or carer (NP: 150); smoking in the same room (NR: 11; NP: 150); Number people in the household (NP: 150); OECD (NR: 12; NP: 150); Living area (NR: 3; NP: 150). All missing data subsequently imputed for this study.

* Based on UK1990 clinical cut‐offs; healthy weight groups include underweight group. For comparison, the weighted prevalence of overweight and obesity at baseline, defined using International Obesity Task Force reference standards (Cole TJ, Bellizzi MC, Flegal KM, et al. Establishing a standard definition for child overweight and obesity worldwide: international survey. BMJ 2000;320(7244):1240–3): overweight—16.1%; and obese—5.0%.

** Qualification abbreviations: HE, higher education; A/AS/S levels, Advanced/Advanced Subsidiary/Scholarship levels; O‐level/GCSE, Ordinary level/General Certificate of Secondary Education.

Between five and 13.99 years of age, 38.1% of the children (*n* = 1221) experienced at least one inpatient admission (23.9% [*n* = 757], 8.7% [*n* = 268], and 5.5% [*n* = 196] had one, two, or three or more admissions, respectively). Of those with at least one admission, 79.8%, 12.0%, and 8.2% were healthy weight, overweight, or obese, respectively, at age five. In contrast, for children with no hospital admissions between five and 13.99 years, 82.9%, 11.2%, and 5.9% were healthy weight, overweight, or obese, respectively, at age five.

In univariable analyses, overweight at age five was not significantly associated with increased hospital admissions (unadjusted RR: 0.92; 95% confidence interval [CI]: 0.72‐1.17); although obesity at age five was suggestive of increased admissions (1.31; 0.96, 1.77) this finding was not statistically significant (*P* = 0.084). The unadjusted RRs for the confounders showed that boys, those with long‐standing illness, with greater daily screen time use, whose mothers were obese or lone parents were all significantly more likely to be admitted, while those whose mothers were educated to diploma or degree level, or living in households with five or more people, were less likely.

In multivariable analyses, associations between weight status and hospital admissions remained non‐significant (adjusted RR, 95% CI: 0.87, 0.68‐1.10 and 1.16, 0.87‐1.54, for overweight and obesity, respectively) (Table 2). Results remained very similar without inclusion of long‐standing illness as a covariate (Table 2). Analyses on children with complete data (*N* = 3058) produced similar results: adjusted RR, 95% CI: 0.89, 0.71‐1.11 and 1.17, 0.88‐1.56, obtained for overweight and obesity, respectively (data not shown in Table).

**Table 2:** Negative binomial regression results for the association between weight status at age five and subsequent hospital admissions

	Unadjusted		Adjusted (a)		Adjusted (b)	
	Rate ratio (95% CI)	*P* value	Rate ratio (95% CI)	*P* value	Rate ratio (95% CI)	*P* value
Weight status age 5 on admissions 5‐13.99 years						
Healthy weight (ref)	1		1		1	
Overweight	0.92 (0.72, 1.17)	0.472	0.87 (0.68, 1.10)	0.239	0.87 (0.68, 1.10)	0.243
Obese	1.31 (0.96, 1.77)	0.084	1.16 (0.87, 1.54)	0.309	1.21 (0.91, 1.60)	0.184
Weight status age 5 on admissions 5‐9.99 years						
Healthy weight (ref)	1		1		1	
Overweight	0.94 (0.73, 1.22)	0.661	0.89 (0.69, 1.16)	0.388	0.90 (0.70, 1.16)	0.400
Obese	1.42 (1.06, 1.90)	0.021	1.24 (0.95, 1.64)	0.118	1.32 (1.00, 1.74)	0.051
Weight status age 5 on admissions 10‐13.99 years						
Healthy weight (ref)	1		1		1	
Overweight	0.90 (0.61, 1.33)	0.596	0.88 (0.61, 1.26)	0.478	0.88 (0.61, 1.26)	0.481
Obese	1.11 (0.71, 1.76)	0.640	1.03 (0.66, 1.61)	0.897	1.06 (0.68, 1.64)	0.807

Table footnote: Results based on imputed data (20 imputed datasets).

Adjustment: (a) all covariates, (b) all covariates excluding longstanding illness

Results from the analyses examining frequency of admissions between five and 9.99 years, and between 10 and 13.99 years, in relation to weight status at age five, revealed that obesity, but not overweight, was significantly associated with admissions from five to 9.99 years in the univariable analysis (unadjusted RR, 95% CI: 1.42, 1.06‐1.90) but not following adjustment for confounding factors (adjusted RR, 95% CI: 1.24, 0.95‐1.64) (Table 2). However, without adjustment for long‐standing illness, borderline significance was reached with children with obesity having higher rates of admission during this period (adjusted RR, 95% CI: 1.32, 1.00‐1.74). Weight status at age five was not significantly associated with admissions from 10 to 13.99 years in univariable or multivariable analyses (eg, adjusted RR, 95% CI: 0.88, 0.61‐1.26 and 1.03, 0.66‐1.61, for overweight and obesity, respectively) (Table 2). As also shown in the table, again, results remained similar without inclusion of long‐standing illness as a covariate.

Children were most commonly admitted for diseases of the respiratory or digestive system, or for injuries (Table 3), with 21%, 29.4%, and 21.1% of the children being admitted for these conditions, respectively. Tabulations of these more common conditions by weight status at age five are shown in Table 4. Frequency of admissions did not differ by baseline weight status (unadjusted *F*‐test *P* values for overall significance: 0.627 for diseases of the respiratory system; 0.104 for diseases of the digestive system; and 0.109 for injuries).

**Table 3:** Reasons* for inpatient admissions from 5 to 13.99 years

Primary Diagnosis**	ICD10 Code	Number of Admissions	Number of Children
Endocrine, nutritional, and metabolic diseases (all codes)	E00‐E90	38	23
Disorders of thyroid gland	E00‐E07	<5	<5
Diabetes mellitus	E10‐E14	23	14
Disorders of endocrine glands	E20‐E35	6	<5
Obesity and other hyperalimentation	E65‐E68	<5	<5
Eating disorders	F50	<5	<5
Sleep disorders	G47	10	10
Diseases of the respiratory system (all codes)	J00‐J99	370	266
Acute tonsillitis	J03	101	94
Chronic diseases of tonsils and adenoids	J35	43	41
Asthma	J45	112	54
Status asthmaticus	J46	<5	<5
Diseases of the digestive system (all codes)	K00‐K93	395	321
Dental caries	K02	194	178
Gingivitis and periodontal diseases	K05	<5	<5
Gastro‐oesophageal disease	K21	<5	<5
Dyspepsia	K30	<5	<5
Hernias—Inguinal	K40	10	8
Hernias—Umbilical	K42	<5	<5
Irritable bowel syndrome	K58	<5	<5
Other functional intestinal disorders	K59	41	37
Injuries*** (all codes)	F10‐F19; S00‐S99; T00‐T65; T71; T74; T75; T79; V01‐V99; W00‐W99; Y00‐Y36; Y90‐Y91	301	265
Injuries of hip and thigh	S70‐S79	8	8
Injuries of knee and lower leg	S80‐S89	23	23
Injuries of ankle and foot	S90‐S99	6	6
Diseases of the musculoskeletal system and connective tissue (all codes)	M00‐M99	83	68
Arthrosis	M15‐M19	<5	<5
Other joint disorders	M20‐M25	28	24

Table footnotes:

* Conditions of interest/most common reasons decided on a‐priori; this is therefore not a list of all diagnoses for admissions from 5‐13.99 years.

** Primary diagnosis taken to be the first non R/Z code for patients with multiple diagnoses recorded in the field at discharge. R codes include symptoms, signs and abnormal clinical and laboratory findings not elsewhere classified; Z codes include factors influencing health status and contact with health services.

*** Restricted to those with emergency method of inpatient admission.

**Table 4:** Admissions by weight status for conditions with greater than 200 children admitted between 5 and 13.99 years of age by weight status at age 5: Weighted percentages (95% CI)—imputed data

		Weight Status at Age 5 (weighted %; 95% CI)		
		Healthy weight	Overweight	Obese
Diseases of the respiratory system	None	81.8;	11.4;	6.8;
	*n =* 3003	80.1, 83.6	10.0, 12.8	5.7, 7.9
	One or more	80.3;	12.9;	6.8;
	*n =* 266	74.2, 86.4	7.2, 18.5	3.7, 9.9
Diseases of the digestive system	None	82.3;	11.1;	6.6;
	*n =* 2948	80.5, 84.0	9.6, 12.6	5.5, 7.7
	One or more	77.1;	14.8;	8.1;
	*n =* 321	71.3, 82.9	9.7, 19.8	4.5, 11.8
Injuries	None	81.4;	11.8;	6.7;
	*n =* 3004	79.6, 83.3	10.3, 13.3	5.7, 7.8
	One or more	84.9;	7.8;	7.3;
	*n =* 265	80.1, 89.8	4.7, 10.9	3.5, 11.0

## 4 DISCUSSION

Contrary to our hypothesis, we observed that clinically defined obesity, or overweight, at primary school entry was not associated with a higher rate of subsequent all‐cause hospital admission up to age 13.99 years or for the three most common reasons for inpatient admissions in this cohort, namely, diseases of the digestive or respiratory system and injuries.

To our knowledge, this is the first study in Wales and Scotland to link childhood weight status measured prospectively in a nationally representative population sample of primary school children to their hospital inpatient admissions. In addition to the strengths of this prospective study design, other strengths include very high‐linkage rates resulting in near complete population‐based coverage of EHRs for these MCS cohort members who were living in Wales and Scotland. This enabled exploration of the timing and reasons for all admissions from five to 13.99 years of age.

We were able to take advantage of measures of height and weight obtained by trained interviewers using standardized protocols in the MCS and, by using published UK1990 reference standards, able to identify children who were “clinically” overweight or obese—that is, children who are defined as being in possible need of clinical intervention.^26^ We did not incorporate BMI measures taken at later ages into our analyses because of the longitudinal nature of the hospital admissions, ranging from age 5 to 14 years. If we had, some of the admissions would have preceded these additional weight measures. We used weight status at 5 years as the exposure of interest because, by this age, most children are already set into their weight centile; nearly 70% of MCS children who were obese at age five maintained this weight status at age 11.^20^

The breadth of information recorded in MCS also enabled consideration of and adjustment for potential confounding factors in the regression analyses. For the latter, use of NBR models accounted for overdispersion of our count data. Other strengths of this study include use of response weights, and multiple imputation methods, to address attrition and missing data, the latter under the assumption that the missing mechanism was MAR.

Linking to administrative health data in Northern Ireland and England was not within scope of this study; however, fieldwork for the current survey of the cohort in 2018/2019, at age 17 years, is obtaining consent for electronic health linkage from all cohort members again, aiding an onward linkage opportunity across the United Kingdom and medical records to be accessed beyond 14 years of age. In turn, this will enable us to repeat our analyses for the whole of the United Kingdom and to determine whether rates of hospitalization increase with age. Our CIs for rates of all‐cause admissions for children with obesity were wide, reflecting limited statistical power. For this reason, our finding of borderline significance for children with obesity having higher rates of admission between 5 and 9.99 years must also be interpreted with caution. Sample size was also insufficient to examine within severe obesity,^32^ which is likely to be associated with more serious acute and chronic health conditions^33^ and with, one might expect, greater health service use. We did not examine other forms of secondary health service use, such as A&E or Outpatient Department (OPD) attendances as these were not available across the full age range considered here. Furthermore, we were unable to capture hospital admissions for children who may have been lost to follow‐up, ie, if they no longer lived in Wales or Scotland or used health services in these countries; in such cases, they were classified as having no hospital admissions beyond that point—we recognize this as a study limitation.

Our findings are consistent with those from Lynch and colleagues^12^ who analysed electronic BMI and health service utilization data for just under 20 000 CYP, aged 2 to 18 years at baseline and living in Minnesota (US); CYP with overweight or obesity were not hospitalized more frequently than those who were a healthy weight, although they did report higher frequency of A&E and OPD visits.

Similarly, analyses of the 2002 to 2005 Medical Expenditure Panel Study (MEPS), a national probability survey in the United States, reported increased utilization of A&E and OPDs by adolescents with obesity and of outpatient clinics by children with obesity.^6^ These findings were confirmed in the later 2005 to 2009 MEPS data.^13^ Children enrolled in the 2001 to 2010 National Health and Nutrition Examination Survey who were overweight attended A&E department but not primary care centres more frequently over a 1‐year period.^34^ These studies are, however, limited by use of parent report of child height and weight^6^, ^13^ or of their child's health care usage.^34^

Hampl and colleagues^14^ reported no significant association between weight status and primary care and A&E visits, for 8404 young people who had measured height and weight at a well‐child clinic and followed up for 12 months. Similarly, Estabrooks and Shetterly^35^ explored billing records for children who received health care in Colorado, United States; they reported a small but significant increase in rates of medical visits in children who were overweight relative to those with healthy weight. Our use of cohort data linked to EHRs from children's use of the NHS, which is free for all UK residents at the point of delivery, overcomes limitations of these studies from the United States that are confined to children integrated into largely private health care systems and therefore only children that used medical services during the study period, thereby limiting their generalizability.

We identified one Canadian study with a similar study design to ours in which a prospective cohort study was linked with administrative health data in Nova Scotia.^8^ Over a five‐year period, 10 to 11 year old children who were obese, but not overweight, visited their physician and had specialist referrals more often than their healthy weight peers. This group also incurred higher health care costs. Consequently, a follow‐up study examined types of disorders experienced; relative to healthy‐weight children, those who were overweight and obese were more likely to have had a diagnosis of asthma (or other respiratory disorder), obesity, otitis media, chronic adenoid/tonsil disorder, and an internalizing disorder (eg, symptoms of depression and anxiety) but not for an infectious disease or injury.^36^ Our study found no significant difference by baseline weight status for the most common reasons for admissions, namely, diseases of the digestive or respiratory system and injuries. Admissions for dental caries were also high, supporting recent concern about increasing numbers of children requiring hospital treatment for this condition,^37^ although we did not examine the association between weight status and dental caries because of low power. The strength of this relationship is unclear in existing studies.^38^

Further research is needed to understand whether our finding changes with increasing age and to examine associations between weight status and A&E and OPD attendances, use of child and adolescent mental health services, and general practitioner consultations. Replication including in larger data sets would enable our findings to be confirmed in relation to hospital admissions or other health‐related outcomes, such as length of time spent in hospital, and to investigate health service use in CYP with severe obesity, and associated comorbidities, and to inform the provision of adequate services and treatment.

In conclusion, our study has shown that clinical obesity at primary school entry is not associated with a higher rate of subsequent hospital admission up to age 14 years. Recent estimates suggest that 4.9% (*n* = 410 000) of 2 to 14 year olds with obesity in England were eligible for secondary care referral;^17^ however, our findings suggest that in a cohort of children born at the beginning of the new millennium, increased admissions in early childhood and adolescence are not directly attributable to childhood obesity. Our findings are, however, not a cause for complacency, given the rising prevalence of obesity in subsequent generations of children born in the United Kingdom after this cohort and the lack of follow‐up into adult life possible currently. Obesity prevention strategies remain key to reducing the prevalence and impact of childhood obesity, and more research is needed to support evidence‐based commissioning of health care services for the management of obesity in CYP.^17^

## CONFLICTS OF INTEREST

No conflict of interest was declared.

## AUTHOR CONTRIBUTIONS

Study design: L.G., M.C‐B., C.D. (all authors commented on study protocol); Data curation and processing: A.A., K.T., A.B., R.A.L., L.G.; Data Analysis: L.G., M.C‐B., B.D.S.; Draft manuscript: L.G., C.D.; Manuscript review: L.G., M.C‐B., K.T., A.B., A.A., B.D.S., N.F., H.B., R.A.L., C.D.; Funding acquisition: C.D. and R.A.L.

## ETHICS APPROVAL

Ethical approval for the fourth survey of the Millennium Cohort Study was received from the Northern and Yorkshire Research Ethics Committee (07/MRE03/32). This study was approved by the Secure Anonymised Information Linkage (SAIL) Information Governance Review Panel (project 232/410) in Wales and the Public Benefit and Privacy Panel for Health and Social Care (project 1617–0160) in Scotland.
